# Management of Aberrant Frenum and Gingival Overgrowth in Orthodontic Patients: A Case Report

**DOI:** 10.7759/cureus.62055

**Published:** 2024-06-10

**Authors:** Akanksha Gupta, Unnati Shirbhate, Priyanka Paul, Pavan Bajaj, Lovely Bharti, Shreeya Panchal

**Affiliations:** 1 Dentistry, Sharad Pawar Dental College, Datta Meghe Institute of Higher Education and Research, Wardha, IND; 2 Department of Periodontics, Sharad Pawar Dental College, Datta Meghe Institute of Higher Education and Research, Wardha, IND; 3 Public Health Dentistry, Sharad Pawar Dental College, Datta Meghe Institute of Higher Education and Research, Wardha, IND; 4 Department of Orthodontics, Sharad Pawar Dental College, Datta Meghe Institute of Higher Education and Research, Wardha, IND; 5 Oral and Maxillofacial Surgery, Sharad Pawar Dental College and Hospital, Datta Meghe Institute of Higher Education and Research, Wardha, IND

**Keywords:** frenectomy, gingival overgrowth, high frenal attachment, gingivoplasty, gingivectomy

## Abstract

The frenum, a fold of mucous membrane, connects the lip and cheek to the alveolar mucosa, gingiva, and underlying periosteum. When the frenum is positioned excessively near the gingival margin, it has the potential to compromise gingival health, impeding plaque control efforts and inducing muscular stress. A frenectomy is a commonly employed corrective measure for anomalous frenum attachments. In a recent clinical case, a 21-year-old female patient was referred from the Department of Orthodontics to the Department of Periodontics due to a papillary-type aberrant labial frenum attachment and excessive gingival tissue surrounding the upper right and left central incisors. The patient underwent a frenectomy, gingivectomy, and gingivoplasty procedures under local anesthesia to address the abnormal frenum attachment and gingival overgrowth using a scalpel. This approach has been demonstrated to yield optimal outcomes in orthodontic therapy for patients exhibiting elevated frenum attachment and gingival overgrowth. Following the achievement of hemostasis, a periodontal pack was applied to facilitate healing and preserve the soft tissue.

## Introduction

Aesthetic purposes have been considered the primary reason motivating people to achieve the perfect smile, which is still the goal in the modern world. In adults, a midline diastema, the permanent absence of contact between the two central incisors in the upper dental arcade, is usually considered an esthetic issue. An abnormally positioned or large frenum is regarded as a significant cause of this issue, so it is essential to pay attention to this structure [1]. Also, where the frenum is situated near the gum line, this has implications on gum health and may lead to gingival recession due to tension from the muscles. Thus, reducing the frenum becomes essential not only from the aesthetic point of view but also to prevent the negative consequences for the general health of the gums [2].

Essential to this examination is the labial frenum, a continuation of fibrous-mucous tissue that attaches the lip to the alveolar mucosa/gingiva and the sub-jacent periosteum. Frenal attachment is categorized based on the extent of muscle fiber attachment: Fibers can be classified as mucosal which is fibers that are attached at the mucogingival junction; gingival where fibers are integrated into the associated gingiva; and papillary where fibers are extended into the interdental papilla. Also, the palatine papilla is affected when the frenum fibers extend over the alveolar process. The following are some of the reasons as to why the frenum needs to be removed. First, an unusual attachment can cause an occurrence of a gap between the front teeth, commonly referred to as midline diastema. Moreover, the tight frenum attached to the gum line may pull the gum flat and pull back the gum line, causing the patient to have a gum receding problem and thus affecting the aesthetic appearance of the smile and oral cavity. Finally, a close frenum connection and a slight oral vestibule lead to several oral health and functional problems that need frenectomy. If diagnosed early enough, these conditions can help deal with issues involving the frenum, and good oral hygiene can also be achieved [3].

The maxillary central incisors may be difficult or impossible to position correctly due to the attached labial frenum. Opinions from different orthodontists on the necessity and timing of labial frenectomies vary. Some suggest that to avoid problems with the ability to close the diastema between the teeth, the frenum be removed as soon as feasible [3,4]. Some advocate performing the frenectomy after the gap has been closed, assuming that the scar tissue will assist in keeping the gap closed. Very rarely does a third group of physicians choose to remove the frenum. Instead, they place bonded retainers on the two central incisors to delay the gap’s potential to widen further. The situation determines which solution is best. How the gingival tissues are arranged and significantly shaped influences how beautiful a set of natural or artificial teeth looks [4]. Patients receiving orthodontic treatment with fixed orthodontic appliances frequently have gum overgrowths [5]. These overgrowths indicate they are more likely to retain plaque, which causes gingival inflammation. However, orthodontic bands might also have drawbacks, like discomfort in the gingiva [6].

## Case presentation

**Figure 1:**
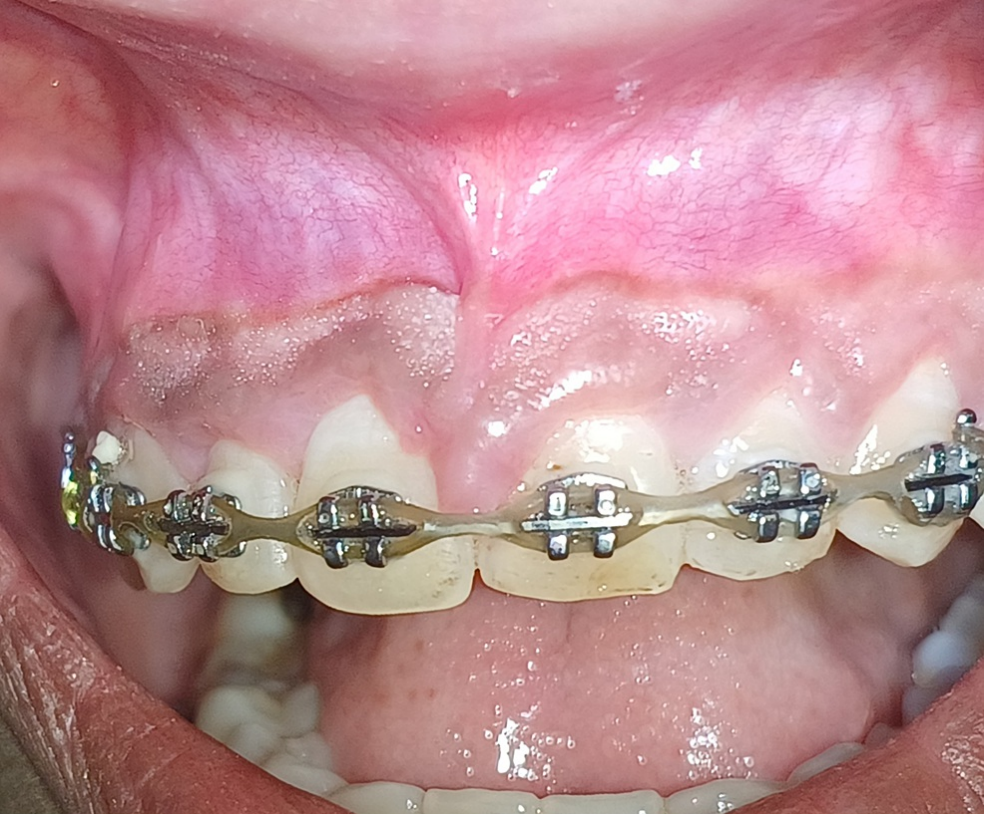
Preoperative view showing high frenal attachment and gingival enlargement with central incisors in orthodontic patient

A 21-year-old female patient reported to the Department of Periodontics, referred from the Department of Orthodontics due to an abnormal frenal attachment and gingival overgrowth associated with the maxillary right and left central incisors, which can be appreciated in Figure 1. Past medical history was not significant. A thorough medical history and periodontal assessment were made, and on clinical examination, the papillary-type frenal attachment was revealed. Hematological investigations were performed and found to be within normal ranges. With no contraindications identified, surgical interventions, comprising frenectomy, gingivectomy, and gingivoplasty were recommended. The patient provided written informed consent before undergoing the surgical procedures, commencing with the surgical procedure.

**Figure 2:**
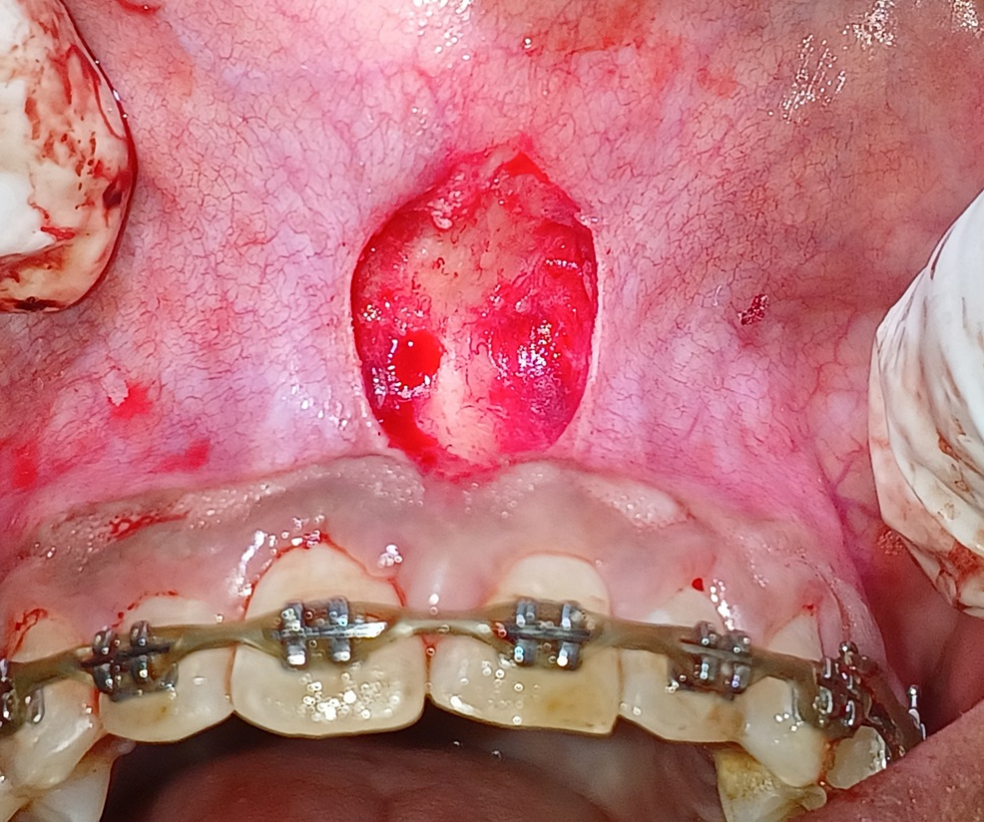
Labial frenectomy was performed with conventional scalpel technique

Under all asepsis and local anesthesia, the conventional scalpel technique where the frenum is positioned till the extent of periosteum and the following steps were performed to remove abnormal labial frenum. The hemostat was positioned between the frenum at the deepest position, after which two incisions were made on the upper and under surfaces of the frenum, and the triangular wedge-shaped tissue was excised, as seen in Figure 2. Following the removal of frenal fibers and undermining of frenal fibers with a scalpel, the frenum assumed a diamond shape, and hemostasis was achieved.

**Figure 3:**
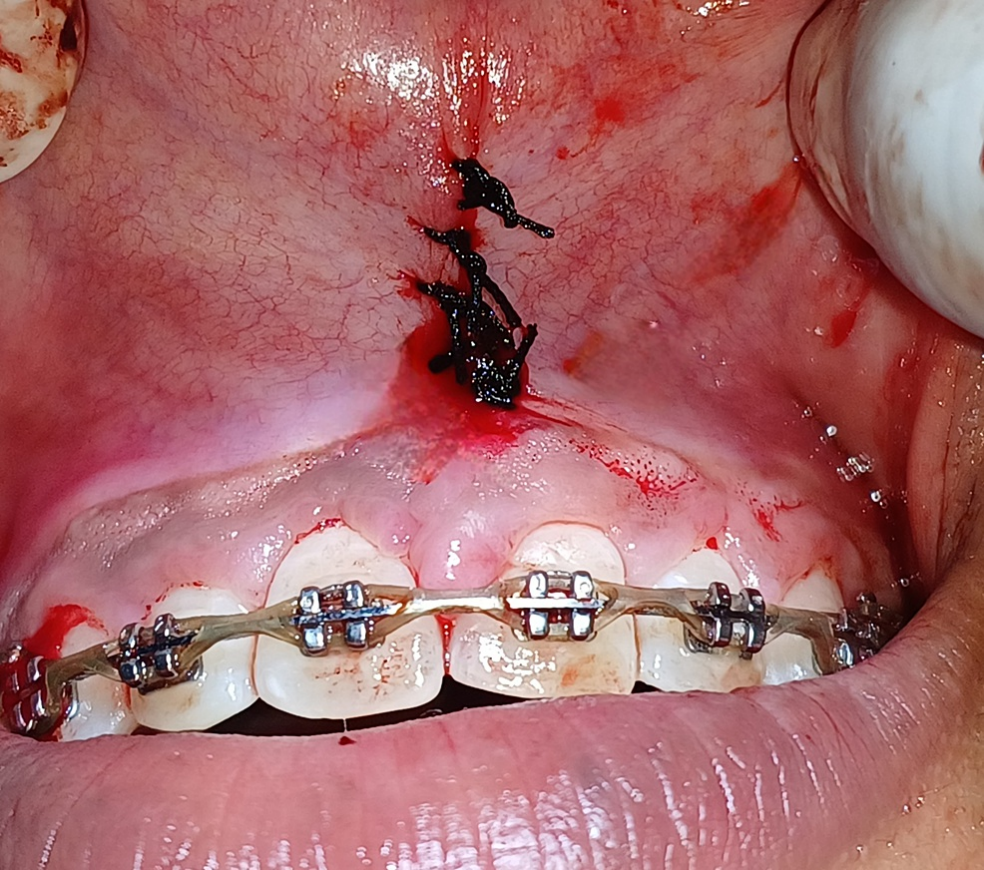
Sutures were placed at the surgical site

After achieving hemostasis, simple interrupted sutures were placed at the surgical site as shown in Figure 3.

**Figure 4:**
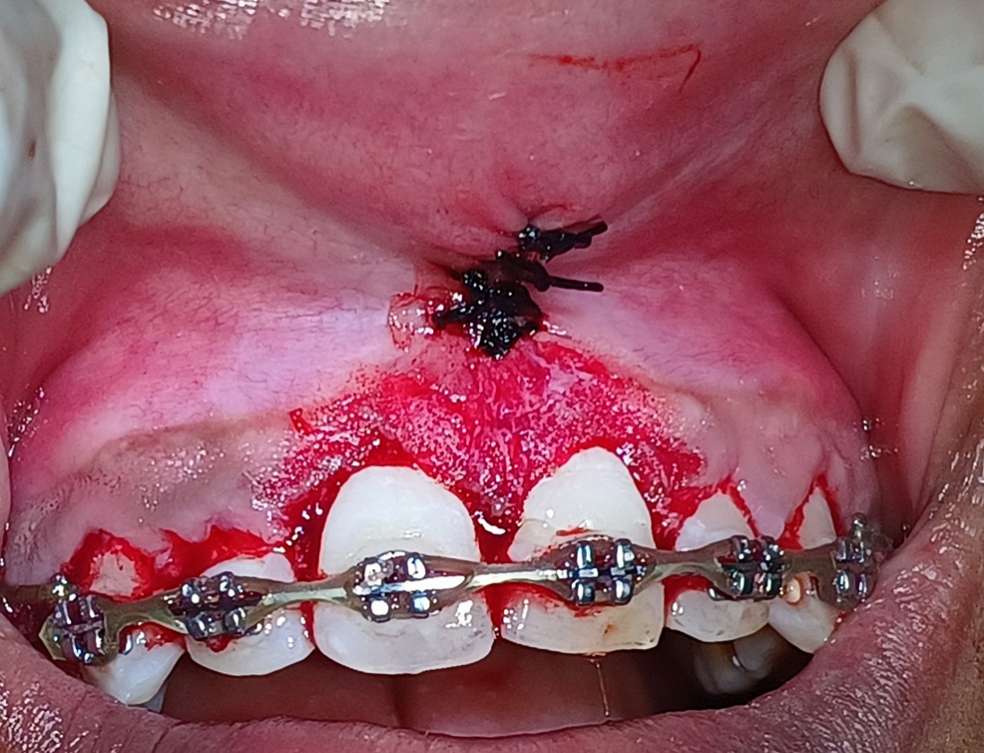
Gingivectomy and gingivoplasty procedure performed with right and left maxillary central incisors

For the management of orthodontic gingival overgrowth associated with right and left central incisors, bleeding points were marked using a pocket marker and a gingivectomy procedure was performed by giving external bevel incision with a conventional scalpel technique followed by gingivoplasty by using periodontal knife to maintain tapering of the gingival margin and scalloped gingival margin, for shaping interdental papillae which are appreciated in Figure 4.

**Figure 5:**
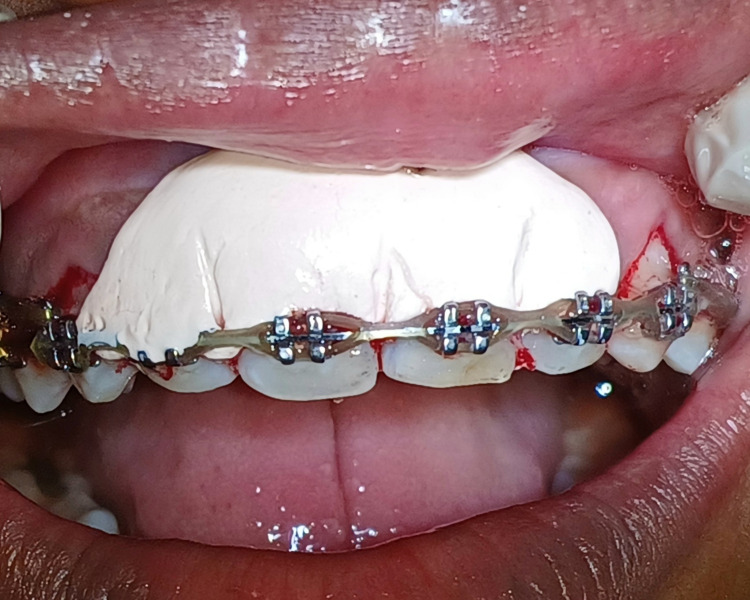
Periodontal pack was placed at the surgical site

After achieving hemostasis, the periodontal pack was applied at the surgical site seen in Figure 5. The sutures were removed after one week showing satisfactory healing and no signs of any infection or discomfort.

**Figure 6:**
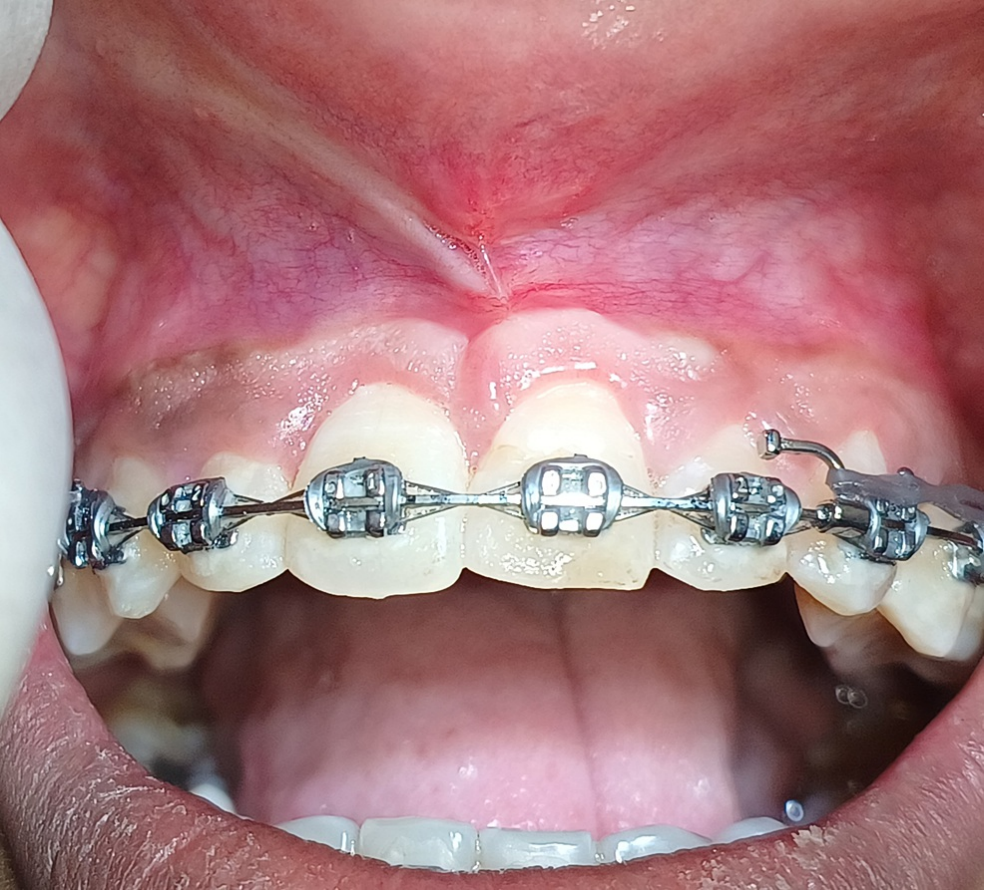
Postoperative view after three months showing complete satisfactory healing and improved esthetics

The patient was reviewed after three months and showed complete satisfactory healing at the surgical site and improved aesthetics. There were no signs of recurrence and minimal scar formation resulting in an aesthetically pleasing outcome appreciated in Figure 6. 

## Discussion

Dentistry has become one of the critical sectors in the present generation that assists in enhancing facial appearance. In particular, the frenum must be stressed since this is one of the major causes of a diastema between the teeth that is still present. In some cases, we can combine gingivoplasty or gingival draft treatment with frenum surgery. If the frenum is sutured close to the gum, this can lead to a condition where the gingiva recedes [7]. It is expected to have the maxillary frenulum attached to the surface of the anterior alveolar ridge, which is related to several periodontal complications. They are the rate at which the patients develop plaque accumulation, diastema, the space between the upper central incisors, gingival recession, and oral health-related issues. Failure to address these issues leads to gingival diseases, aesthetically compromised teeth, and, in some instances, tooth loss [8]. This technique was recommended for patients with midline diastema with an abnormally positioned frenum to eliminate the muscle fibers connecting the palatine papilla with the orbitalis oris [2]. Since an abnormally positioned frenum is one of the reasons for the persistence of midline diastema, it is noticed that it is necessary to pay attention to the frenum. Among such procedures that are referred to as frenectomy or frenotomy, there are interventions aimed at helping to deal with an abnormally located frenum [9,10].

Some people have recommended that lasers and electrosurgery could be the alternatives to frenectomies conducted using an ordinary technique and a scalpel. The results of this method are precise, and they are different depending on the technique used; the method can be used to control tissue accurately and achieve quicker healing, but there are also some disadvantages of the process; for instance, it can lead to the formation of long-term incisions that may cause gum diseases and aesthetic issues [11]. Scar formation should be discussed and treated, particularly when the scar interferes with the closure of the midline gap due to orthodontic work. As a result, one should avoid the removal of frenum before orthodontic interventions in cases of midline diastema. Based on esthetics and periodontal diseases, an integrated approach is required to manage cases of abnormally positioned frenum and overgrown gum tissues in orthodontic patients. Frenum anomalies cause midline diastema and gum recession and, as a result, affect both the smile’s esthetic appearance and periodontal health [11,12]. The two surgical procedures, gingivectomy and gingivoplasty, resulted in physiologic and esthetic gingival contour and eradication of plaque retentive factors. To address these issues, several options exist, including a frenectomy and a gingivectomy, with the option selected based on the patient’s preference and that of the clinician [12,13].

Drawbacks for the current frenectomy technique may include discomfort to the patient who are fearful of dental treatment, sometimes minimal scar formation will be there, pain, and delayed wound healing sometimes but due to suturing the wound will get healed by primary intention [10,12]. Devishree et al. reported that while treating an aberrant frenum with modification techniques functional and an aesthetic outcome can be achieved by a proper technique selection [3]. Bajaj et al. demonstrated desirable outcomes, with Z-plasty providing aesthetic clearance in the orthodontic treatment procedure despite the complex treatment procedure [12]. Shirbhate et al. reported V-Y plasty being a reliable option for covering the defects caused by frenectomy and elongating the structures with better clinical outcomes [11].

## Conclusions

Orthodontic therapies combined with periodontal surgical intervention to achieve the best result and outcome while treating orthodontic patients. By careful evaluation, the surgical protocol should be carried out. In the following case presentation, the combined approach of frenectomy and gingivectomy in the esthetic region resulted in improvement using the conventional scalpel technique in functional and esthetic characteristics. Clinicians can improve orthodontic patients’ aesthetic appearance and periodontal health by contributing to their general well-being and satisfaction with the smile esthetics.
